# A Novel Inhibitor of Human La Protein with Anti-HBV Activity Discovered by Structure-Based Virtual Screening and *In Vitro* Evaluation

**DOI:** 10.1371/journal.pone.0036363

**Published:** 2012-04-27

**Authors:** Jing Tang, Zhi-Min Huang, Ying-Yi Chen, Zhao-Hui Zhang, Gao-Lin Liu, Jian Zhang

**Affiliations:** 1 Department of Pharmacy, First People's Hospital Affiliated with Shanghai JiaoTong University, Shanghai, China; 2 Department of Pathophysiology, Key Laboratory of Cell Differentiation and Apoptosis, Chinese Ministry of Education, Shanghai JiaoTong University School of Medicine, Shanghai, China; 3 Department of Urology, Ruijin Hospital Luwan Branch Affiliated with Shanghai JiaoTong University School of Medicine, Shanghai, China; University of Cincinnati College of Medicine, United States of America

## Abstract

**Background:**

Over 350 million people worldwide are infected with hepatitis B virus (HBV), a major cause of liver failure and hepatocellular carcinoma. Current therapeutic agents are highly effective, but are also associated with development of viral resistance. Therefore, strategies for identifying other anti-HBV agents with specific, but distinctive mechanisms of action are needed. The human La (hLa) protein, which forms a stabilizing complex with HBV RNA ribonucleoprotein to promote HBV replication, is a promising target of molecular therapy.

**Aims:**

This study aimed to discover novel inhibitors of hLa that could inhibit HBV replication and expression.

**Methods:**

A multistage molecular docking approach was used to screen a Specs database and an in-house library against hLa binding sites. Sequential *in vitro* evaluations were performed to detect potential compounds with high scores in HepG2.2.15 cells.

**Results:**

Of the 26 potential compounds with high scores chosen for experimental verification, 12 had HBV DNA inhibition ratios of less than 50% with *P*<0.05. Six had significant inhibition of HBV *e* antigen (HBeAg) levels, and 13 had significant inhibition of HBV surface antigen (HBsAg) levels by *in vitro* assays. Compounds HBSC-11, HBSC-15 and HBSC-34 (HBSC is system prefix for active compounds screened by the library) were selected for evaluation. HBSC-11 was found to have an obvious inhibitory effect on hLa transcription and expression.

**Conclusions:**

Our findings suggest that anti-HBV activity of HBSC-11 may be mediated by a reduction in hLa levels. In addition, our data suggest the potential clinical use of hLa inhibitors, such as HBSC-11, for treating HBV infection.

## Introduction

More than 350 million people worldwide are chronically infected by the hepatitis B virus (HBV), increasing their risk of developing chronic hepatitis, liver cirrhosis, and hepatocellular carcinoma [1]. Antiviral resistance and side effects have limited the utility of currently approved therapies. HBV, a member of the hepadnaviridae family, is a non-cytopathic virus with a 3.2 kb circular DNA genome. After conversion into covalently closed circular DNA in the host cell nucleus, this genome serves as a template for transcription of all viral RNAs [2].

The potential of small RNA interference to induce post-transcriptional down-regulation of HBV RNA in a stable HBV-expressing cell line, HepG2.2.15, has been highlighted in the past [3]. The human La (hLa) protein, in concert with additional trans-acting factors, forms an HBV RNA ribonucleoprotein complex stabilizing HBV RNA [2] by binding at a site that is located within the viral RNA between nucleotides 1275 and 1291 [4], [5]. Protein hLa is a 47 kDa phosphoprotein predominantly localized in the nucleus, and was originally identified as a major target of the autoimmune response in patients suffering from the autoimmune diseases Sjogren's syndrome and systemic lupus erythematosus [6]. Since then, it has been characterized as a conserved RNA-binding protein that interacts specifically with oligouridylate stretches in the RNA [7].

The hLa protein has been shown to be associated with all RNA polymerase III transcripts that carry the UUU_OH_ sequence as the transcription termination signal. Therefore, this protein is believed to play a central role in the metabolism of these RNAs, possibly acting as a molecular chaperone to stabilize and support RNAs for further processing [8]. In addition, the hLa protein has also been suggested to stabilize various other critical human RNAs, such as histone, and RNAs of pathogens that have taken up residence in the host cell, such as hepatitis C and B virus [4], [9], [10].

Among the different La proteins identified in a variety of organisms, the N-terminal sequence is highly conserved [11]. The three-dimensional (3D) structure of the hLa-RNA domain was determined by X-ray crystallography [7]. In addition, studies have elucidated the structures of hLa-RNA complex with the highly conserved N-terminus, the La motif, the central RNA recognition motifs (RRM), the C-terminal RRM (NRE), and the important serine residue (Ser) 366 [6], [7].

The objective of this study was to identify novel classes of compounds capable of inhibiting hLa and determine their potential for blocking HBV replication and expression.

## Results

### Computational modeling and virtual screening

**Figure 1:**
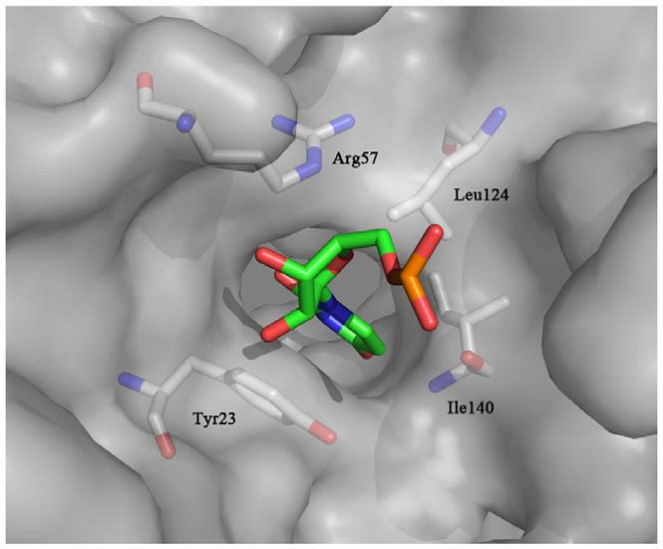
RNA binding pocket of the human La protein. All crystallographic water molecules were removed from the coordinate set, and missing resides were repaired in the Maestro v7.5 molecular modeling platform. Oxygen atoms are shown in red and nitrogen atoms in blue. Carbon atoms of RNA are shown in green. Carbon atoms of protein are shown in gray. Key residues of RNA binding sites are shown as sticks and are labeled. Figures were generated by the PyMol molecular visualization system (www.pymol.org).

Grid-based ligand docking from energetics (GLIDE) software [12] (Schrodinger, Portland, OR) was used for computational modeling and virtual screening based on the X-ray crystal structure of the human La-RNA complex [7] (2VOD in the Protein Data Bank, 2.1 Å). In preparation for docking, RNA was removed and only one of the two symmetrical monomers (chain A) was used as template (Figure 1). To validate the docking approach, the U-2 nucleotide, which bound most deeply into the RNA groove of the La protein, was extracted from the crystal structure of chain A and used to perform docking. As expected, the optimal docking pose closely reproduced the X-ray crystal structure (data not shown). Three-dimensional structures of compounds from the Specs chemical library were downloaded and processed with LigPrep software (obtained from Schrodinger). Each chemical structure (for small molecules) was docked into the U-2 nucleotide binding site using GLIDE 5.5, and the Glide scoring function (G-Score) was used to rank these final poses for all compounds in decreasing order. The binding poses of the top 30 compounds were stored for visual inspection of the docking geometry to avoid unreasonable interactions. Finally, 26 of the 30 compounds were selected for evaluation of inhibition of HBV and effects on hLa expression.

### Biological evaluations

**Figure 2:**
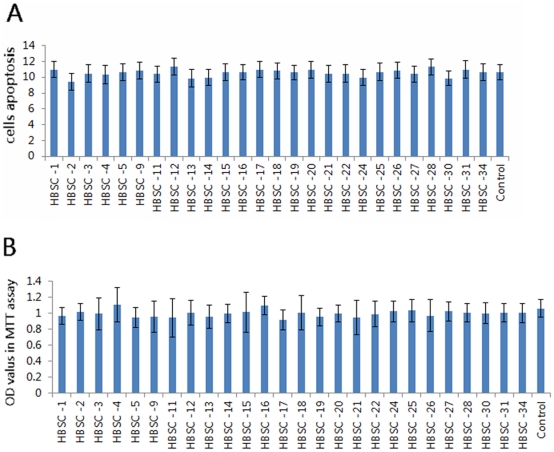
Cell viability assays after exposure to candidate compounds. Changes in cell numbers were measured by MTT assays. Cells were collected after 24 h of treatment with 50 µM inhibitors. Induction of cell apoptosis (rh Annexin V/FITC) was determined according to the manufacturer's instructions. Differences in (A) apoptosis or (B) surviving cell numbers were observed 24 h after treatment with HBSC compounds compared to controls.

**Figure 3:**
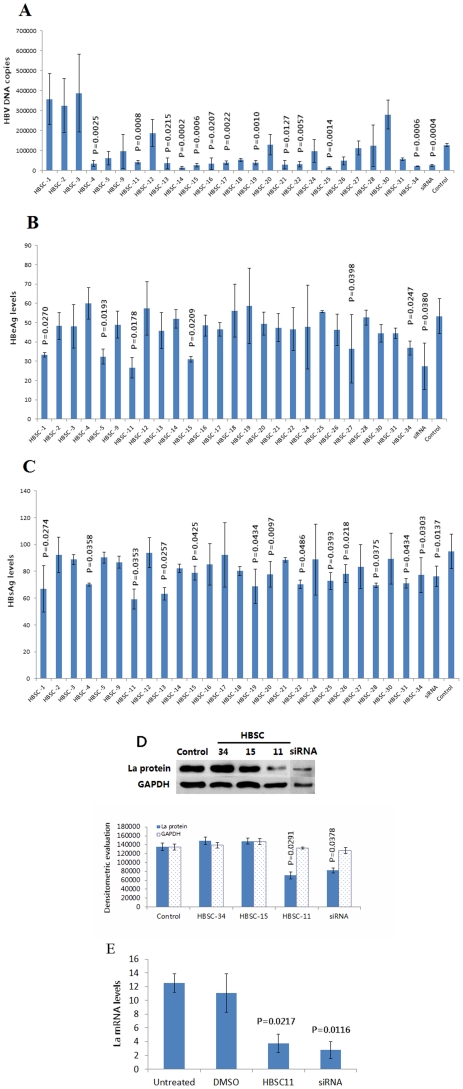
*In vitro* evaluations of HBSC compounds. HBV DNA levels were detected by FQ-PCR, and HBeAg and HBsAg levels were analyzed by MEIA assays. hLa transcription and expression were detected by qRT-PCR and Western Blotting. (A) 12 compounds exhibited active inhibition of HBV DNA replication and (B) 6 compounds exhibited active inhibition of HBeAg expression and (C) 13 compounds decreased the levels of HBsAg expression and (D) 1 compound (HBSC-11) showed significant inhibition of hLa protein levels on Western blots. (E) 1 compound (HBSC-11) down-regulated the hLa mRNA levels, as compared with that produced by the siRNA.

To screen the anti-HBV activity of the 26 potential compounds from the database, we measured their capacities to inhibit HBV DNA replication and HBV *e* antigen (HBeAg) and HBV surface antigen (HBsAg) levels in the HepG2.2.15 cell line. This well-established cell line is permanently transfected with a plasmid containing two head-to-tail dimers of the HBV genome, and constitutively expresses HBV genes. Therefore, cultured HepG2.2.15 cells can not only release high levels of HBsAg and HBeAg into the media, but also support the assembly and secretion of replicative intermediates of HBV DNA and Dane particles [13]. Baseline virion DNA levels in cell culture supernatants were measured to confirm that the HepG2.2.15 cells were stably expressing HBV particles. The potential effect of HBSC compounds on cell viability was evaluated by MTT assays, and flow cytometry. Little difference in apoptosis or surviving cell numbers was observed 24 h after treatment with HBSC compounds when compared to controls (Figures 2A and B). Results showed that the average quantity of HBV DNA was 10^6^ copies/mL and HBsAg and HBeAg reactivity was strongly positive. From the screening, 12 compounds were found to be capable of inhibiting HBV DNA replication with ratio less than 50% (Figure 3A: HBSC-4 *vs* Control = 27.18% *vs* 100%, *P* = 0.0025; HBSC-11 *vs* Control = 32.59% *vs* 100%, *P* = 0.0008; HBSC-13 *vs* Control = 27.75% *vs* 100%, *P* = 0.0215; HBSC-14 *vs* Control = 11.06% *vs* 100%, *P* = 0.0002; HBSC-15 *vs* Control = 20.44% *vs* 100%, *P* = 0.0006; HBSC-16 *vs* Control = 27.06% *vs* 100%, *P* = 0.0207; HBSC-17 *vs* Control = 30.06% *vs* 100%, *P* = 0.0022; HBSC-19 *vs* Control = 31.20% *vs* 100%, *P* = 0.0010; HBSC-21 *vs* Control = 22.37% *vs* 100%, *P* = 0.0127; HBSC-22 *vs* Control = 25.20% *vs* 100%, *P* = 0.0057; HBSC-25 *vs* Control = 11.71% *vs* 100%, *P* = 0.0014; HBSC-34 *vs* Control = 17.07% *vs* 100%, *P* = 0.0006; siRNA *vs* Control = 20.62% *vs* 100%, *P* = 0.0004), while 6 compounds resulted in significantly decreased levels of HBeAg (Figure 3B: HBSC-1 *vs* Control = 62.57% *vs* 100%, *P* = 0.0270; HBSC-5 *vs* Control = 60.59% *vs* 100%, *P* = 0.0193; HBSC-11 *vs* Control = 49.89% *vs* 100%, *P* = 0.0178; HBSC-15 *vs* Control = 58.04% *vs* 100%, *P* = 0.0209; HBSC-27 *vs* Control = 68.35% *vs* 100%, *P* = 0.0389; HBSC-34 *vs* Control = 69.05% *vs* 100%, *P* = 0.0247; siRNA *vs* Control = 51.37% *vs* 100%, *P* = 0.0380) and 13 compounds resulted in significantly decreased the levels HBsAg (Figure 3C: HBSC-1 *vs* Control = 70.60% *vs* 100%, *P* = 0.0274; HBSC-4 *vs* Control = 73.96% *vs* 100%, *P* = 0.0358; HBSC-11 *vs* Control = 62.52% *vs* 100%, *P* = 0.0353; HBSC-13 *vs* Control = 66.69% *vs* 100%, *P* = 0.0257; HBSC-15 *vs* Control = 83.02% *vs* 100%, *P* = 0.0425; HBSC-19 *vs* Control = 72.62% *vs* 100%, *P* = 0.0434; HBSC-20 *vs* Control = 81.97% *vs* 100%, *P* = 0.0097; HBSC-22 *vs* Control = 73.97% *vs* 100%, *P* = 0.0486; HBSC-25 *vs* Control = 76.75% *vs* 100%, *P* = 0.0393; HBSC-26 *vs* Control = 82.50% *vs* 100%, *P* = 0.0218; HBSC-28 *vs* Control = 73.41% *vs* 100%, *P* = 0.0375; HBSC-31 *vs* Control = 74.96% *vs* 100%, *P* = 0.0434; HBSC-34 *vs* Control = 81.41% *vs* 100%, *P* = 0.0303; siRNA *vs* Control = 80.58% *vs* 100%, *P* = 0.0137).

Of the 26 selected compounds, only HBSC-11, HBSC-15 and HBSC-34 were found to significantly decrease HBeAg, HBsAg and HBV DNA levels (Figures 3A–C). Therefore, these three compounds were selected to further evaluate their inhibition of hLa levels in HepG2.2.15 cells. Finally, only one compound, HBSC-11, was shown to have significant inhibitory effects on hLa expression, as evidenced by Western blotting (Figure 3D, HBSC-11 *vs* Untreated cells = 20.42% *vs* 100%, *P* = 0.0069; siRNA *vs* Untreated cells = 28.59% *vs* 100%, *P* = 0.0302) and qRT-PCR (Figure 3E, HBSC-11 *vs* Untreated cells = 29.73% *vs* 100%, *P* = 0.0217; siRNA *vs* Untreated cells = 19.42% *vs* 100%, *P* = 0.0116).

### Binding mode analyses of HBSC compounds

**Figure 4:**
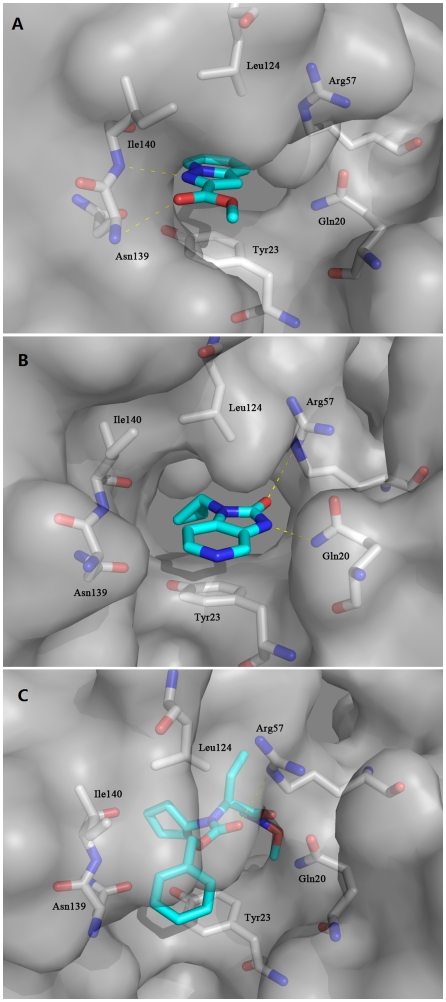
Predicted binding modes of HBSC-11 (A), HBSC-15 (B) and HBSC-34 (C) compounds to the human La protein. The protein surface is shown in gray. Oxygen atoms are shown in red and nitrogen atoms in blue. Carbon atoms of HBSC compounds and protein are shown in cyan and gray, respectively. Crucial residues in the binding site are shown as sticks and labeled. Hydrogen bonds between HBSC compounds and protein are depicted as yellow dotted lines. Figures were generated by PyMol.

**Figure 5:**
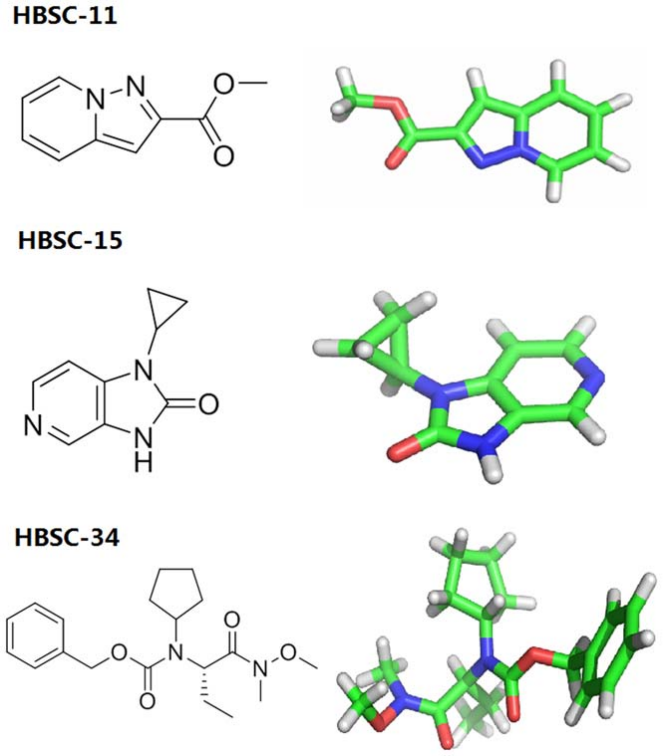
Chemical formulas and space structures of selected compounds. Carbon atoms are shown in green. The hydrogen atom is shown in white. Oxygen atoms are shown in red and nitrogen atoms in blue. Compounds are shown as sticks and are labeled.

To gain insight into the mechanism by which HBSC compounds affect the biological function of the La protein, we carried out docking simulation to evaluate binding of the HBSC compounds to human La protein. The results showed that HBSC compounds enter a pocket composed of Gln20, Tyr23, Arg57, Leu124, Asn139 and Ile140 (Figure 4A). HBSC-11 was found to form two hydrogen bonds with the nitrogen atom of the main chain of Asn139 and Ile140. Figure 4B shows that HBSC-15 formed two hydrogen bonds with the nitrogen atom of the side chains of Gln20 and Arg57. Figure 4C shows that HBSC-34 formed one hydrogen bond with the nitrogen atom of the side chain of Arg57. The deduced chemical formula and spatial structure of these compound candidates are shown in Figure 5. Typically, favorable functional groups such as sulfonyl, carboxyl and hydroxyl were found in all 26 of the best scoring compounds from the screening process.

## Discussion

Currently, two major classes of agents are utilized in the therapy of HBV infection: the interferon and the nucleoside analogues [14], both of which are effective for viral suppression, but do not result in high rates of sustained viral response. In addition, the emergence of drug-resistant HBV strains may limit the long-term therapeutic potential of nucleoside analogues. Therefore, the development of novel antiviral drug targets is warranted.

The La protein is a multifunctional RNA-binding protein, which has been implicated in the stabilization of HBV RNA [4]. La binds to a small RNA element [2], [4], [5] embedded in the posttranscriptional regulatory element [15] that protects viral RNA against endoribonucleolytic cleavage events [10]. In the case of HBV, hLa in concert with additional transacting factors form an HBV RNA ribonucleoprotein complex that stabilizes HBV RNA [2]. Previous studies have demonstrated that La depletion by RNA interference negatively affects HBV expression [16] and down regulates HBV mRNA levels in HepG2.2.15 cells, which supports the concept that La plays a role in the HBV viral life cycle [17]. It is possible that La may be a potential drug target for controlling HBV gene expression.

Virtual screening can serve as a valuable tool in the identification of novel effective inhibitors [18]. Although no similar computational approach has been reported in the literature for identifying inhibitors of La in HBV, there have been many reports on inhibitors of hepatitis C virus nonstructural protein 3 (HCV NS3) helicase/protease that have been identified by virtual screening using the program DOCK [19]–[21].

Here, for the first time, we have demonstrated the feasibility of using X-ray crystallographic data and computational modeling as a basis for a structure-based virtual screening to identify chemical inhibitors of La. Our novel strategy used large-scale structure-based computational analysis followed by a series of *in vitro* evaluations. On the basis of structural analysis of hLa, two separate RRM binding sites were targeted. Both sites have known competitive inhibitors. Moreover, RRM has been characterized as an efficient submicromolar competitive inhibitor of this hLa. In addition, the structural similarity of the inhibitors to the RRM strongly suggests their ability to interact with the RRM pocket. Therefore, on the basis of the specific structural interactions of RRM, we sought inhibitory candidates directed against these target sites.

Our virtual screening approach was based on initial high-throughput docking calculations that were performed on a library of more than 300,000 commercially available compounds. Using a personal computer (PC) grid to harness the computing power of our university's PCs, we were able to perform these calculations without prior focusing of the compound library. After the compounds had been docked, compound poses were refined and promising candidates were assayed *in vitro*. Insights from these assays, combined with pharmacophoric searches based on the predicted binding site, were then used to select further compounds for follow-up testing.

Amino acid residues located within 12 Å from Tyr23 in the center of the RNA groove of the La protein were defined as part of the binding site for docking studies. However, the RNA binding site is rather shallow and solvent-exposed, so that molecules interacting firmly with this site are difficult to find. The second site binds the C-terminal RRM, which invokes problems of specificity and off-target activity. Searching for new La-RNA binding site inhibitors, we carried out a virtual screening campaign and identified a La-RNA binding complex as the most potent and optimizable hit. Until now, there have been no reports on a La autoantigen specific inhibitor. Therefore, no proper control was available to identify the function of new inhibitors. To solve this problem, we chose the compounds with high scores (calculated from the Glide software) to identify inhibitors with the highest potential for targeting the La-RNA binding site. We utilized hLa siRNA to compare the inhibition capacity of the selected compound as our previous research [16].

From the viral expression assays, three compounds that exhibited significant decreases in HBV DNA, HBeAg and HBsAg levels were selected for further study. We analyzed the structure of these three compounds, and found that two of them had similar core structures and complicated three-domain structures. Among the three compounds, only one, HBSC-11, appeared to have a siRNA effect, as evidenced by significantly reduced La levels in treated HepG2.2.15 cells. HBSC-11 is a a pyrazolo and pyridine compound with the chemical name of methyl pyrazolo [1,5-a] pyridine-2-carboxylate. This is a low molecular weight compound with a simple structure that is easy to synthesize. Further study on derivatives of this compound is warranted.

In conclusion, using virtual high-throughput screening, we have discovered a new La inhibitor. Cell viability and siRNA assays indicated that HBSC-11 is well accommodated. Docking modeling revealed a potential binding pocket adjacent to the La-RNA site in the RRM domain. Moving away from the endogenous binding pocket allows for identification of residues that are less conserved and may induce some selectivity among La inhibitors. These observations have led us to investigate a new series of HBSC-11 derivatives and open the door for discovery of additional specific inhibitors targeting La.

## Materials and Methods

### Protein preparation

The crystal structure of human La protein, previously determined by Curry *et al.* [7], was retrieved from the Protein Data Bank (PDB entry: 2VOD). Residues located within 12 Å from Tyr23 in the center of the RNA groove of the La protein were defined as the binding pocket for docking studies. All crystallographic water molecules were removed from the coordinate set, and missing resides were repaired using the Maestro v7.5 molecular modeling platform (Schrodinger, Inc.) [22].

### Grid-based ligand docking from energetics (GLIDE)

Glide calculations were performed with Maestro v7.5. Hydrogen atoms and charges were added during a brief relaxation that was performed using the Protein Preparation module in Maestro with the “preparation and refinement” option. A restrained partial minimization was terminated when the root-mean-square deviation (rmsd) reached a maximum value of 0.3 Å, in order to relieve steric clashes. The grid-enclosing box was centered on the oxygen atom of the side chain of Tyr23 and defined so as to enclose residues located within 14 Å from the center of RNA groove. A scaling factor of 1.0 was set to the van der Waals (VDW) radii of the receptor atoms with partial atomic charges of less than 0.25. In the docking process, standard precision (SP) and extra precision (XP) docking were respectively adopted to generate the minimized pose, while the Glide scoring function (G-Score) was used to select the final three poses for each ligand. The docking simulations were performed in parallel on a Dell Cluster server.

### Virtual screening

Virtual screening of large chemical databases is a successful approach for lead identification. Here, both Specs database (http://www.specs.net/) and an in-house generated compound library were employed to identify potential inhibitors of hLa.

### In vitro evaluation

#### Cell culture

The human hepatoblastoma HepG2.2.15 [13], a stable HBV-expressing cell line, was maintained in our lab according to the method described by Zhang *et al* [16]. Briefly, cells were grown in Dulbecco's modified Eagle's medium (DMEM) (Gibco BRL, Gaithersburg, MD, USA) supplemented with 0.03 g/L L-glutamine, 10% heat-inactivated fetal calf serum (FCS) (Gibco BRL, Gaithersburg, MD, USA) , 100 U/mL penicillin, and 100 µg/mL streptomycin (Gibco BRL, Gaithersburg, MD, USA) at 37°C in a humidified atmosphere containing 50 mL/L CO_2_. For experiments, 348 µg/mL G418 sulfate (Sigma-Aldrich, St. Louis, MO, USA) was added to the medium. To assess cell growth and viability, equal volumes of cell suspension and 0.4% trypan blue solution were mixed and placed on a hemocytometer for counting. Only cells with a viability of >85% were used for further analysis.

#### Evaluation of potential inhibitors

Inhibitors were dissolved in dimethyl sulfoxide (DMSO) (<0.5% volume of total medium; Amresco, Solon, OH, USA) and then sterilized by filtration through a 0.22 µm filter. Cells were treated for 24 h with inhibitors at final concentrations of 50 µM each. All cell treatments were conducted in triplicate, and control cells were treated with equal amounts of the DMSO solvent alone under identical conditions.

#### Cell viability assays

The changes in cell numbers were detected by 3-(4, 5)-dimethylthiahiazo (-z-y1)-3, 5-di- phenytetrazoliumromide (MTT) (Amresco, Solon, OH, USA) assay after 24 h exposure to various inhibitors or vehicle controls. Plates were incubated at 37°C and 5% CO_2_ in a humidified cell culture incubator. MTT (final concentration of 0.5 mg/mL) was added to each well at 21 h and incubation continued for 3 h, after which 0.2 mL of acidified isopropanol was added to each well to dissolve the MTT formazan [23]. Each experiment was repeated in triplicate. Absorbance of MTT formazan was measured at 572 nm and 690 nm. The corrected absorbance was determined by subtracting the 690 nm value from the 572 nm value. Cells were collected after 24 h of treatment with 50 µM inhibitors, and induction of cell apoptosis (rh Annexin V/FITC) was determined according to the manufacturer's instructions (Beijing 4A Biotech Co. Ltd., Beijing, China).

#### Detection of HBV expression

After treatment with inhibitors for 24 h, culture supernatants of HepG2.2.15 cells were collected. Extracellular HBV DNA was assayed by fluorogenic quantitative polymerase chain reaction (FQ-PCR), according to the manufacturer's instructions (Da An Gene Co. Ltd., Sun Yat-sen University, Guangzhou, China). The assay was capable of quantifying HBV DNA with a theoretical lower limit of detection of 5 genome equivalents per mL of supernatant. The extracellular levels of HBV HBsAg and HBeAg produced from HepG2.2.15 cells were measured using the ELISA kit according to the manufacturer's instructions (Beijing Bioneovan Co.,Ltd, Beijing, China). Samples were considered reactive for HBsAg or HBeAg, respectively, if the values of the sample rate/index calibrator mean rate (S/N) was greater than or equal to 2.0, and value of the sample rate/cut off rate (S/CO) was greater than or equal to 2.1.

#### siRNA assay

According to the hLa mRNA sequence (NM003142) from GenBank, three hLa mRNA-specific siRNAs (siRNA1, siRNA2 and siRNA3) were designed using the web-based tools from www.qiagen.com/siRNA and BLAST. A negative control siRNA (siRNA4) was constructed with the same nucleotide composition as the experimental siRNA but which lacked significant sequence homology to the hLa, and its sequence was confirmed by BLAST. Synthesis of siRNAs by *in vitro* transcription of DNA templates was performed with the Silencer® siRNA construction kit (Ambion, Austin, TX, USA), and the transfection experiment was carried out with the RNAiFect transfection reagent (Qiagen, Hilden, Germany). An electrochemiluminescence method was used to detect HBeAg and HBsAg levels in the supernatants of cells after siRNAs treatment [16]. From the above screening method, the most effective siRNA was selected and transfected into HepG2.2.15 cells.

#### Western blotting and real time reverse transcription (qRT-) PCR analysis

HepG2.2.15 cells were harvested from 15 cm plates (Falcon; BD Biosciences, Franklin Lakes, NJ, USA) by using lysis buffer containing 20 mM Tris (pH 7.5), 150 mM NaCl, 1 mM EDTA, 1 mM EGTA, 1.0% Triton-X-100, 0.5% Nonidet P-40, 2.5 mM sodium pyrophosphate, 1 mM Na_3_VO_4_, 1 mg/ml leupeptin, and 1 mM PMSF. Lysates were sonicated, and protein concentrations were determined by BCA assay. Proteins were resolved by 12% SDS-PAGE and transferred to PVDF membranes (Roche Applied Science, Penzberg, Germany) [24]. Membranes were blocked with 5% nonfat milk in Tris-buffered saline Tween-20 (TBST) for 1 h, followed by an overnight incubation at 4°C with the primary antibody of anti-hLa (1∶1000 in 1% nonfat milk, Abnova Corp., Taiwan). The next day, membranes were washed with TBST and then incubated for 2 h with the secondary antibody of horseradish peroxidase (HRP)-conjugated goat anti-mouse (1∶1000; Bio-Rad, Hercules, CA, USA) at room temperature. Afterwards, the membranes were washed with TBST and immunoreactive bands were detected by incubating with enhanced chemilluminescence reagents (ECL; Amersham Biosciences, Piscataway, NJ, USA). Bands were visualized by exposure to X-ray film (Eastman Kodak Co., Rochester, NY, USA), which was converted to a digital image using a CanoScan N650U scanner (Cannon, New Holland, PA, USA). Images were visualized in Adobe Photoshop CS.

For qRT-PCR analysis, isolated RNA (starting material, pellets, and supernatants) was dissolved in 43 µL of diethyl pyrocarbonate-treated water. Then, 5 µL of RNase-free DNase reaction buffer and 2 units of RNase-free DNase (Promega, Madison, WI, USA) were added, and the mixture was incubated for 30 min at 37°C. DNase was inactivated by heating the sample for 5 min at 72°C. Two µL of DNase-treated RNA was applied as template for qRT-PCR. qRT-PCR was conducted with the Titan One-Tube qRT-PCR kit (Roche Applied Science) and the following primer pairs: La sense, 5′-TGCTGTTTGTGAGCCTGTGGCG-3′, and anti-sense, 5′-TCCCGTGGCAAATTGAAGTCGC-3′; β-actin sense, 5′-AGAGCCTCGCCTTTGCCGATCC-3′, and anti-sense, 5′-CTGGGCCTCGTCGCCCACATA-3′. qRT-PCR was performed with the same primers as those for standard RT-PCR but on a LightCycler with Fast Start RNA Master SYBR Green I reagents (Roche Diagnostics, Basel, Switzerland). The thermal cycling protocol included an RT reaction at 61°C for 20 min, followed by denaturation at 95°C for 30 s and 40 amplification cycles of 95°C for 5 s, 60°C for 30 s, and 72°C for 10 s with a single fluorescence measurement. The melting curve program was set at 60–95°C with a heating rate of 0.1°C per s with continuous fluorescence measurement and a final cooling step to 40°C. Negative control experiments that omitted the RT step or used water instead of RNA yielded no products (data not shown). Integrity of all reactions was confirmed by demonstration of product melting curves with a melting point of 83°C and negligible nonspecific products. In addition, gel electrophoresis and ethidium bromide staining of aliquots of qRT-PCR products revealed that correct-sized bands were the only products.

#### Statistical analysis

Results are presented as mean±SEM. Statistical significance were analyzed using the Student's *t*-test for two groups and one-way analysis of variance for multi-group comparisons. A *P*-value<0.05 was considered statistically significant.

## References

[1] Lee WM (1997). Hepatitis B virus infection.. N Engl J Med.

[2] Ehlers I, Horke S, Reumann K, Rang A, Grosse F (2004). Functional characterization of the interaction between human La and hepatitis B virus RNA.. J Biol Chem.

[3] Heise TD, Dandri UM, Petersen J, Rang A, Burda MR, Dienes HP, Schirmacher P, Brechot K, Brechot K, Okuda K (2000). In Chronic Hepatitis: New Concepts of Pathogenesis, Diagnosis and Treatment.

[4] Heise T, Guidotti LG, Chisari FV (1999). La autoantigen specifically recognizes a predicted stem-loop in hepatitis B virus RNA.. J Virol.

[5] Horke S, Reumann K, Rang A, Heise T (2002). Molecular characterization of the human La protein hepatitis B virus RNA.B interaction in vitro.. J Biol Chem.

[6] Teplova M, Yuan YR, Phan AT, Malinina L, Ilin S (2006). Structural basis for recognition and sequestration of UUUOH 3′ temini of nascent RNA polymerase III transcripts by La, a rheumatic disease autoantigen.. Molecular Cell.

[7] Kotik-Kogan O, Valentine ER, Sanfelice D, Conte MR, Curry S (2008). Structural analysis reveals conformational plasticity in the recognition of RNA 3′ ends by the human La protein.. Structure.

[8] Schäffler K, Schulz K, Hirmer A, Wiesner J, Grimm M (2010). A stimulatory role for the La-related protein 4B in translation.. RNA.

[9] Spangberg K, Wiklund L, Schwartz S (2001). Binding of the La autoantigen to the hepatitis C virus 3′ untranslated region protects the RNA from rapid degradation in vitro.. J Gen Virol.

[10] Heise T, Guidotti LG, Chisari FV (2001). Characterization of nuclear RNases that cleave hepatitis B virus RNA near the La protein binding site.. J Virol.

[11] Maraia RJ, Intine RV (2001). Recognition of nascent RNA by the human La antigen: conserved and diverged features of structure and function.. Mol Cell Biol.

[12] Friesner RA, Banks JL, Murphy RB, Halgren TA, Klicic JJ (2004). Glide: a new approach for rapid, accurate docking and scoring. 1. Method and assessment of docking accuracy.. J Med Chem.

[13] Sells MA, Chen ML, Acs G (1987). Production of hepatitis B virus particles in HepG2 cells transfected with cloned hepatitis B virus DNA.. Proc Natl Acad Sci U S A.

[14] Stein LL, Loomba R (2009). Drug targets in hepatitis B virus infection.. Infect Disord Drug Targets.

[15] Donello JE, Beeche AA, Smith GJ, Lucero GR, Hope TJ (1996). The hepatitis B virus posttranscriptional regulatory element is composed of two subelements.. J Virol.

[16] Zhang H, Sun JH, Geng HL, Fan LY, Liu GL (2006). A study on the relativity between La protein and the stability of HBV mRNA and the expression of HBV protein.. Zhonghua Gan Zang Bing Za Zhi.

[17] Ni Q, Chen Z, Yao HP, Yang ZG, Liu KZ (2004). Inhibition of human La protein by RNA interference downregulates hepatitis B virus mRNA in 2.2.15 cells.. World J Gastroenterol.

[18] Melagraki G, Afantitis A (2011). Ligand and structure based virtual screening strategies for hit-finding and optimization of hepatitis C virus (HCV) inhibitors.. Curr Med Chem.

[19] Chen CS, Chiou CT, Chen GS, Chen SC, Hu CY (2009). Structure-based discovery of triphenylmethane derivatives as inhibitors of hepatitis C virus helicase.. J Med Chem.

[20] Talele TT, Arora P, Kulkarni SS, Patel MR, Singh S (2010). Structure-based virtual screening, synthesis and SAR of novel inhibitors of hepatitis C virus NS5B polymerase.. Bioorg Med Chem.

[21] Talele TT, Arora P, Kulkarni SS, Patel MR, Singh S (2011). Discovery of novel HCV polymerase inhibitors using pharmacophore-based virtual screening.. Bioorg Med Chem Lett.

[22] Glide, version 5.5, Schrödinger, LLC, New York, NY, 2009

[23] van de Loosdrecht AA, Beelen RH, Ossenkoppele GJ, Broekhoven MG, Langenhuijsen MM (1994). A tetrazolium-based colorimetric MTT assay to quantitate human monocyte mediated cytotoxicity against leukemic cells from cell lines and patients with acute myeloid leukemia.. J Immunol Methods.

[24] Hoke SM, Liang G, Mutiu AI, Genereaux J, Brandl CJ (2007). C-terminal processing of yeast Spt7 occurs in the absence of functional SAGA complex.. BMC Biochem.

